# Estimating cranial musculoskeletal constraints in theropod dinosaurs

**DOI:** 10.1098/rsos.150495

**Published:** 2015-11-04

**Authors:** Stephan Lautenschlager

**Affiliations:** School of Earth Sciences, University of Bristol, Life Sciences Building, 24 Tyndall Avenue, Bristol BS8 1TQ, UK

**Keywords:** Dinosauria, functional morphology, musculature, muscle strain, digital reconstruction

## Abstract

Many inferences on the biology, behaviour and ecology of extinct vertebrates are based on the reconstruction of the musculature and rely considerably on its accuracy. Although the advent of digital reconstruction techniques has facilitated the creation and testing of musculoskeletal hypotheses in recent years, muscle strain capabilities have rarely been considered. Here, a digital modelling approach using the freely available visualization and animation software Blender is applied to estimate cranial muscle length changes and optimal and maximal possible gape in different theropod dinosaurs. Models of living archosaur taxa (*Alligator mississippiensis*, *Buteo buteo*) were used in an extant phylogenetically bracketed framework to validate the method. Results of this study demonstrate that *Tyrannosaurus rex*, *Allosaurus fragilis* and *Erlikosaurus andrewsi* show distinct differences in the recruitment of the jaw adductor musculature and resulting gape, confirming previous dietary and ecological assumptions. While the carnivorous taxa *T. rex* and *Allo. fragilis* were capable of a wide gape and sustained muscle force, the herbivorous therizinosaurian *E. andrewsi* was constrained to small gape angles.

## 1. Introduction

The anatomy, size and arrangement of the jaw muscles are important functional factors underpinning an animal’s capability for vocalization [1], social signalling [2–4] and—most importantly—food acquisition (i.e. capture, manipulation, processing) [5–7]. These factors determine jaw closing and opening speeds, jaw closing force (=bite force), jaw gape, and horizontal and vertical jaw movements, thereby constraining food selection and prey or food size [8,9], feeding behaviour [4,10–12] and the occupation of ecological niches [13,14]. In extinct animals, these parameters are often difficult to determine. Soft-tissue structures are rarely preserved and information on myological structures has to be inferred from preserved hard tissues (osteological correlates) or by comparison with extant taxa, which either form a phylogenetic bracket or a functional analogue [15,16]. As such soft-tissue reconstructions lie at the base of functional inferences, accurate reconstructions are paramount for wider ecological or behavioural deductions in extinct vertebrates.

Recent advances in digital imaging techniques have enabled detailed studies of the cranial musculature in living vertebrates [17–19], providing a steadily increasing catalogue of extant anatomical data. Similarly, novel approaches for three-dimensional, digital muscle reconstructions have provided detailed models and musculoskeletal hypotheses for extinct taxa [20,21]. In contrast with traditional descriptions and two-dimensional reconstructions, the digital nature of this data permits not only the extraction and quantification of muscle properties (length, physiological cross-section area, volume), but also the functional analysis of cranial systems [22–24]. Among the growing number of these studies, though, the maximal possible strain (or excursion) of cranial muscles is rarely considered and the few exceptions [25] do not take three-dimensional morphology into account. Similarly, studies on (fossil) taxa implementing muscle information have focused on locomotory function and the measurement of moment arms [26–28] rather than muscle excursion. However, muscles can only stretch a certain amount before they tear, thus limiting the maximum gape angle and jaw opening. Furthermore, muscular performance is closely related to the extension of muscle fibres [29–31]. Detailed information on these factors can, therefore, provide a better understanding on the feeding behaviour of extinct organisms and complement existing analyses.

Cranial function of theropod dinosaurs has been extensively studied in the past based on direct inferences through tooth marks and feeding traces [32,33], theoretical considerations derived from functional measurements [34,35], and most recently by using computational biomechanical analyses [36–38]. Yet, debate remains regarding the feeding behaviour of different theropods [32,39]. Furthermore, different feeding styles have been suggested ranging from puncture-and-pull feeders (e.g. *Tyrannosaurus rex*) to strike-and-tear feeders (e.g. *Allosaurus* *fragilis*) to secondarily herbivorous, specialized forms (e.g. *Erlikosaurus andrewsi*) [37,40,41]. Here, the musculoskeletal constraints imposed on the crania of selected theropod dinosaurs are evaluated supported by an extant phylogenetically bracketed approach. By applying a novel approach to estimate maximum muscle strain using the three-dimensional modelling and animation software Blender, digital models of *T. rex*, *Allo. fragilis* and *E. andrewsi* are studied to test the hypothesis that musculoskeletal constraints reflect different feeding behaviours and dietary adaptations.

## 2. Material and methods

### 2.1 Digital models: extant taxa

To obtain information about muscle behaviour in extant archosaurs and to provide an extant phylogenetically bracketed framework for the fossil taxa, digital models of a common buzzard (*Buteo* *buteo*) [18] and an alligator (*Alligator mississippiensis*) [42] were used. These taxa were selected as accurate data on muscle architecture and morphology had been gained in previous studies employing contrast-enhanced computed tomography (CT) scanning [17,18].

### 2.2 Digital models: theropod dinosaurs

**Figure 1.**
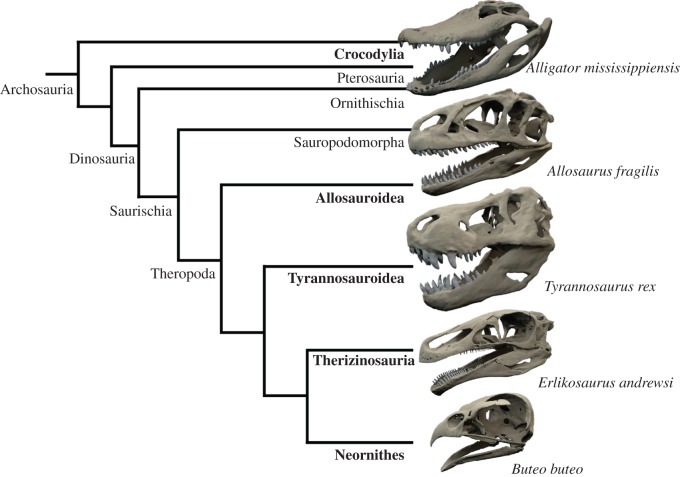
Digital models of the studied fossil theropod and extant archosaur taxa in a simplified phylogenetic context. Cranial models not to scale.

Digital models of the carnivorous theropods *T*. *rex* and *Allo*. *fragilis*, as well as the derived herbivorous theropod *E*. *andrewsi* were used (figure 1) to represent a variety of different feeding modes and dietary specializations among theropods. The digital model of *T. rex* was based on a museum quality cast (of BHI 3033, Black Hills Institute, South Dakota) housed at the Sauriermuseum Aathal, Switzerland, and was digitized using a photogrammetry approach [43] and Agisoft Photoscan Standard (www.agisoft.ru). As no internal features, such as pneumatic cavities or bone microstructure, were relevant for this study, photogrammetry proved to be an effective digitization method. Existing digital models of *Allo*. *fragilis* (MOR 693, Museum of the Rockies, Bozeman) [40] and *E*. *andrewsi* (IGM 100/111, Geological Institute of the Mongolian Academy of Sciences, Ulaan Baatar, Mongolia) [44] were based on X-ray CT scans.

### 2.3 Functional analysis

Blender (www.blender.org) is freely available three-dimensional modelling and visualization software, which allows the creation, modification and animation of digital models. It has occasionally been used in palaeontological studies to create static images and animations for publications [45,46], and also provides the possibility for customized analytical approaches and automatization using the in-built python interpreter as presented here.

Digital models of all taxa were imported into Blender (version 2.71) as ‘.ply’ files with the skull and mandible defined as separate components. Model size was kept at approximately 200 000 elements for the skull models and 100 000 elements for the mandible models (values were chosen so that all models were of high enough resolution to recognize anatomical details, but still small enough to allow for good render performance). The centre of rotation for the mandible was positioned at the jaw joint to allow a realistic opening and closing. The degree of rotation representing the gape angle was manually set to an experimentally determined maximum value (60.0° for *Alli*. *mississippiensis* and *B*. *buteo*; 80.0° for *T*. *rex* and *E. andrewsi*; 90.0° for *Allo*. *fragilis*) which captured a large range of jaw positions (including the gape at maximum tension limit). Rotation was controlled for the duration of a jaw opening cycle using Blender's keyframe animation tool. The duration of the cycle was chosen so that one frame corresponded to 0.5° in order to get sufficient resolution for the muscle strain measurements.

**Figure 2.**
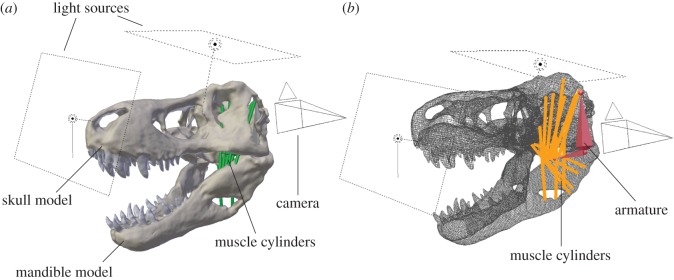
Model and analysis set-up in Blender exemplified for *Tyrannosaurus* *rex* shown in (*a*) solid and (*b*) wireframe view.

Jaw adductor muscles for the studied taxa were modelled as cylinders selected from the in-built geometry primitives library. The individual cylinders were positioned to connect corresponding muscle origin and insertion sites (tables 1 and 2) when the jaw was in a fully closed position (0°) based on previously published muscle reconstructions [15,17,18,20,40]. The cylinders were subsequently connected to an armature consisting of two bone elements originating from the jaw joint and attaching to the ends of the cylinders, in order to allow the extension of the cylinders parallel to the rotation of the mandible (figure 2). For muscles with an extensive origin or insertion, at least two cylinders were used to model the rostral- and caudalmost extent of the muscle, as it has been shown experimentally that individual parts of a single muscle body can have different strain factors [47,48].

**Table 1.** Muscle origins and insertions for extant archosaur taxa used in this study. (Muscle abbreviations: m. AMEM, m. adductor mandibulae externus medialis; m. AMEP, m. adductor mandibulae externus profundus; m. AMES, m. adductor mandibulae externus superficialis; m. AMP, m. adductor mandibulae posterior; m. PSTp, m. pseudotemporalis profundus; m. PSTs, m. pseudotemporalis superficialis; m. PTd, m. pterygoideus dorsalis; m. PTv, m. pterygoideus ventralis.)

	origin	insertion
*Alligator mississippiensis*		
mAMES	rostrolateral surface of quadrate and quadratojugal	dorsolateral surface surangular
mAMEM	rostromedial surface of quadrate	dorsolateral surface of coronoid eminence
mAMEP	ventrolateral surface of parietal	dorsomedial surface of coronoid eminence
mAMP	rostrolateral surface of quadrate	medial mandibular fossa
mPSTs	lateral surface of prootic, rostrolateral surface of parietal	rostral portion of medial mandibular fossa via cartilago transiliens
mPSTp	lateral surface of epipterygoid and prootic	caudodorsal edge of angular
mPTd	dorsal surface of palatine, pterygoid and ectopterygoid	caudomedial surface of angular and articular
mPTv	ventral surface of pterygoid and quadrate	caudoventral surface of angular
*Buteo buteo*		
mAMES	temporal fossa, caudal surface of postorbital process, lateral surface of squamosal	dorsolateral surface of coronoid process
mAMEM	temporal fossa, caudal surface of postorbital process, lateral surface of squamosal	dorsolateral surface of coronoid process
mAMEP	rostral surface of quadrate and otic process	lateral surface of surangular
mAMP	ventral surface of quadrate, otic and mandibular process	dorsomedial surface of surangular and articular
mPSTs	ventral surface of laterosphenoid buttress	processus pseudotemporalis, caudomedial surface of coronoid process
mPSTp	rostrolateral surface of the orbital process of the quadrate	rostromedial surface of medial mandibular fossa
mPTd	dorsal surface of palatine shelf, rostral surface of pterygoid	caudomedial surface of medial mandibular fossa
mPTv	ventral and caudoventral surface of palatine shelf	ventral surface of medial mandibular process, lateroventral surface of articular

**Table 2.** Muscle origins and insertions for fossil theropod taxa used in this study. (Muscle abbreviations as in table 1.)

	origin	insertion
*Allosaurus fragilis*		
mAMES	medial and ventral surface of supratemporal bar on postorbital and squamosal	dorsolateral surface of surangular
mAMEM	medial surface of supratemporal bar on postorbital and squamosal	dorsomedial surface of surangular
mAMEP	caudomedial surface of supratemporal fossa on parietal and suqamosal	dorsomedial surface of surangular
mAMP	lateral surface of quadrate flange	caudomedial surface of medial mandibular fossa
mPSTs	rostral surface of supratemporal fossa on postorbital, parietal and laterosphenoid	rostromedial surface of medial mandibular fossa
mPSTp	lateral surface of laterosphenoid, basisphenoid and pterygoid region	rostromedial surface of medial mandibular fossa
mPTd	dorsal surface of pterygoid, lateral surface of ectopterygoid	medial surface of angular and articular ventral to jaw joint
mPTv	caudoventral surface of pterygoid	lateral and ventral surface of articular and surangular
*Tyrannosaurus rex*		
mAMES	caudomedial surface of supratemporal bar	dorsolateral surface of surangular
mAMEM	caudal surface of supratemporal fossa on squamosal	dorsomedial surface of surangular
mAMEP	caudomedial surface of supratemporal fossa on parietal and sagittal crest	dorsomedial surface of surangular
mAMP	lateral surface of quadrate	medial mandibular fossa
mPSTs	rostral surface of supratemporal fossa on postorbital, parietal and laterosphenoid	rostromedial surface of medial mandibular fossa
mPSTp	lateral surface of laterosphenoid, basisphenoid and pterygoid region	rostromedial surface of medial mandibular fossa
mPTd	dorsal surface of pterygoid, lateral surface of ectopterygoid	medial surface of angular and articular
mPTv	caudoventral surface of pterygoid	lateral and ventral surface of articular and surangular
*Erlikosaurus andrewsi*		
mAMES	medial surface of supratemporal bar on postorbital and squamosal	dorsolateral surface surangular
mAMEM	caudal surface of supratemporal fossa on squamosal/parietal bar	dorsomedial surface of surangular
mAMEP	caudomedial surface of supratemporal fossa on parietal	coronoid eminence
mAMP	lateral surface of quadrate	medial mandibular fossa
mPSTs	rostral surface of supratemporal fossa on postorbital, parietal and laterosphenoid	rostral portion of medial mandibular fossa
mPSTp	lateral surface of laterosphenoid, basisphenoid and pterygoid region	medial mandibular fossa
mPTd	dorsal surface of pterygoid	medial surface of angular and articular ventral to jaw joint
mPTv	caudoventral surface of pterygoid	lateral and ventral surface of articular and surangular

A python script was created for each taxon to measure the strain of each muscle cylinder, to calculate muscle strain ratios between relaxed and stretched muscle states and to export all recorded parameters to a text file for post-processing (see the electronic supplementary material). Additionally, an algorithm was created to colour-code the muscle cylinders according to the extent of muscle stretch for easy visualization. A graphical output for each keyframe was saved as a ‘.jpg’ image and these images were combined into a movie file (see the electronic supplementary material). The python script was written to run for the duration of a complete cycle, but alternatively a stop command can be activated to interrupt the cycle when a specific muscle strain factor is reached. Given the length–tension relationship of muscles, the resting length of cranial muscles has to lie at a small gape angle over 0° in order to generate the necessary force during biting [30,49]. As there are no experimental results at which gape angle resting length occurs, three different analyses were performed for the extant taxa (*Alli*. *mississippiensis*, *B. buteo*) with resting length set at an angle of 3.0°, 6.0° and 9.0°. Results then informed the settings for the theropod taxa, for which the analyses were run with resting lengths at 3.0° and 6.0°. A resting length set at 9.0° turned out to produce strain factors substantially lower than in the extant taxa and were therefore not considered (see Results and Discussion sections for details).

## 3. Results

### 3.1 Extant taxa

**Figure 3.**
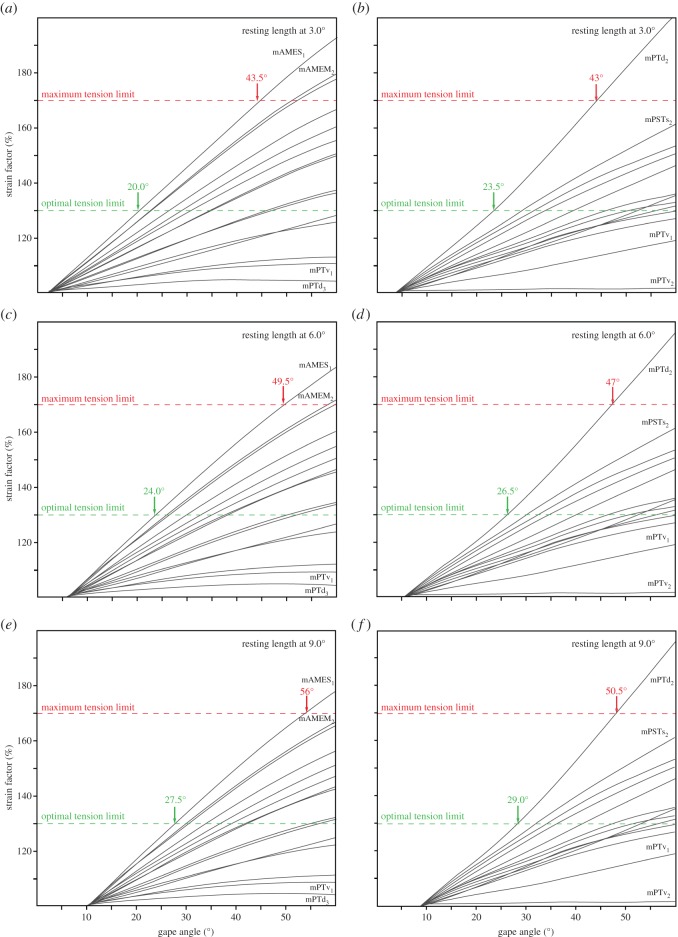
Muscle strain factors plotted against gape angle for (*a*,*c*,*e*) *Alligator* *mississippiensis* and (*b*,*d*,*e*) *Buteo* *buteo*. Analysis were run with resting length set at a gape angle of (*a*,*b*) 3.0°, (*c*,*d*) 6.0° and (*e*,*f*) 9.0°. Muscle abbreviations as in table 1.

Strain analyses were performed for *Alli*. *mississippiensis* and *B*. *buteo* with the muscle resting length set at a gape angle of 3.0°, 6.0° and 9.0°. The plotted ratios between stretched and relaxed muscle lengths (=*strain* factor) against gape angle show a consistent pattern for each taxon regardless of resting length (figure 3). However, owing to the changed resting length, the strain values at 60.0° (maximum gape angle for each analysis) are highest with the resting length at a low gape (3.0°) and, respectively, lowest at higher gape (9.0°). For *Alli*. *mississippiensis* strain factors range from 103 to 193% (resting length at 3.0°) to 102 to 175% (resting length at 9.0°; figure 3*a*,*c*,*e*). Values are somewhat higher for *B*. *buteo* with strain between 101 and 204% (resting length at 3°) and, respectively, between 100 and 188% (resting length at 9°; figure 3*b*,*d*,*f*).

**Figure 4.**
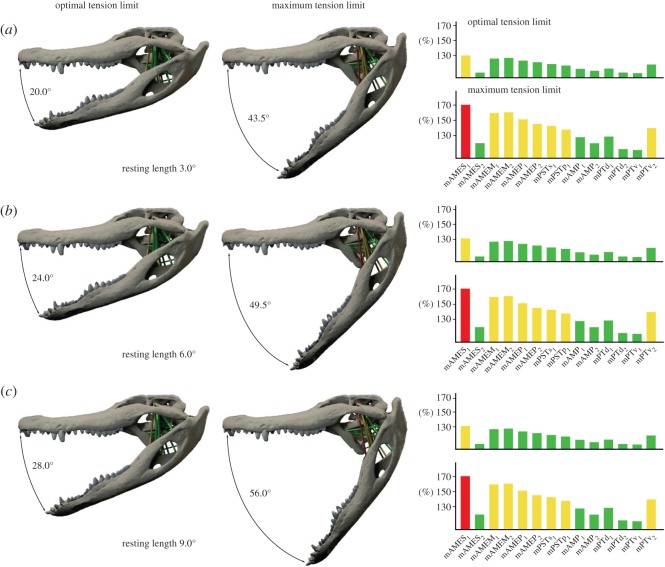
Gape angles at optimal and maximum tension limit for *Alligator* *mississippiensis* with muscle resting lengths at a gape angle of (*a*) 3.0°, (*b*) 6.0° and (*c*) 9.0°. Bar diagrams show strain factors of individual muscles at optimal and maximum tension limit. Muscle abbreviations as in table 1.

**Figure 5.**
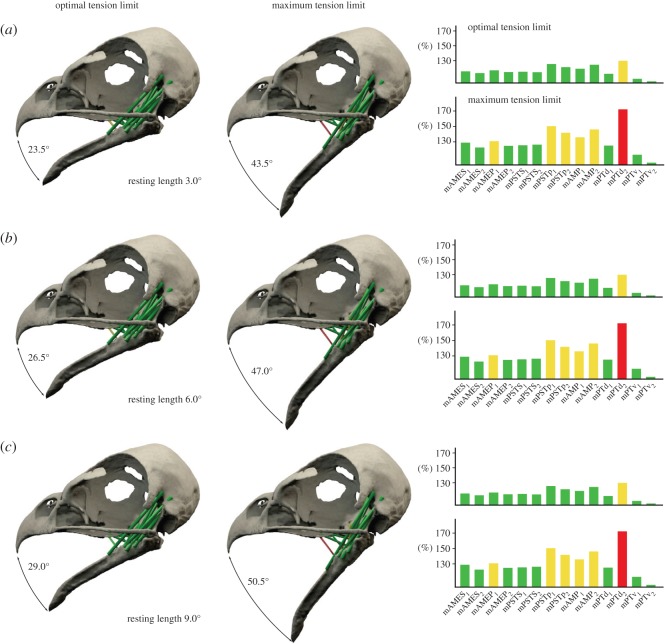
Gape angles at optimal and maximum tension limit for *Buteo buteo* with muscle resting lengths at a gape angle of (*a*) 3.0°, (*b*) 6.0° and (*c*) 9.0°. Bar diagrams show strain factors of individual muscles at optimal and maximum tension limit. Muscle abbreviations as in table 1.

Owing to their internal structure consisting of overlapping filament cross-bridges, muscles have a strain range in which maximal tetanic contraction can be achieved (optimal tension limit, 100–130% of resting length) and a maximum tension limit (170% of resting length) above which contraction is no longer possible [30,31]. When these limits are applied to the strain results for the extant taxa in this study, the optimal tension limit would be reached at gape angles of 20.0°, 24.0° and 27.5° and a maximum tension limit at a gape of 43.5°, 49.5° and 56.0° (for resting lengths at 3.0°, 6.0° and 9.0°) in *Alli. mississippiensis* (figures 3*a*,*c*,*e* and 4). For *B*. *buteo*, the optimal tension limit is reached at a gape of 23.5°, 26.5° and 29.0° and the maximum tension limit at gape angles of 43.0°, 47.0° and 50.5° (for resting lengths at an angle of 3.0, 6.0 and 9.0°; figures 3*b*,*d*,*f* and 5).

### 3.2 Theropod dinosaurs

**Figure 6.**
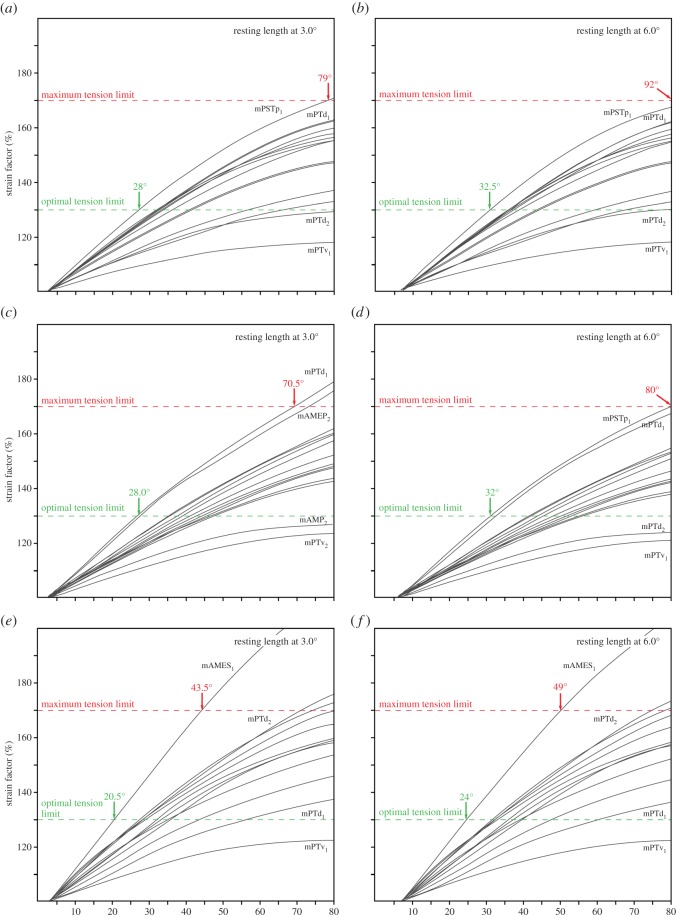
Muscle strain factors plotted against gape angle for (*a*,*b*) *Allosaurus fragilis*, (*c*,*d*) *Tyrannosaurus rex* and (*e*,*f*) *Erlikosaurus andrewsi*. Analysis were run with resting length set at a gape angle of (*a*,*c*,*e*) 3.0° and (*b*,*d*,*f*) 6.0°. Muscle abbreviations as in table 1.

Strain ratios for the theropod taxa were recorded with resting lengths set at a gape angle of 3.0° and 6.0°. As found for the extant taxa, the strain patterns are unchanged for the different resting lengths (figure 6), but strain value changes according to resting length. At a gape angle of 80.0° strain factors are recorded between 117 and 170% (resting length at 3.0°) and between 116 and 164% (resting length at 6.0°) for *Allo*. *fragilis* (figure 6*a*,*b*). For *T*. *rex* similar values are found ranging from 122 to 172% (resting length at 3.0°) and from 121 to 170% (resting length at 6.0°; figure 6*c*,*d*). Strain factors for *E*. *andrewsi* are considerably higher at 122 to 216% (resting length at 3.0°) and 121 to 205% (resting length at 6.0°; figure 6*e*,*f*).

**Figure 7.**
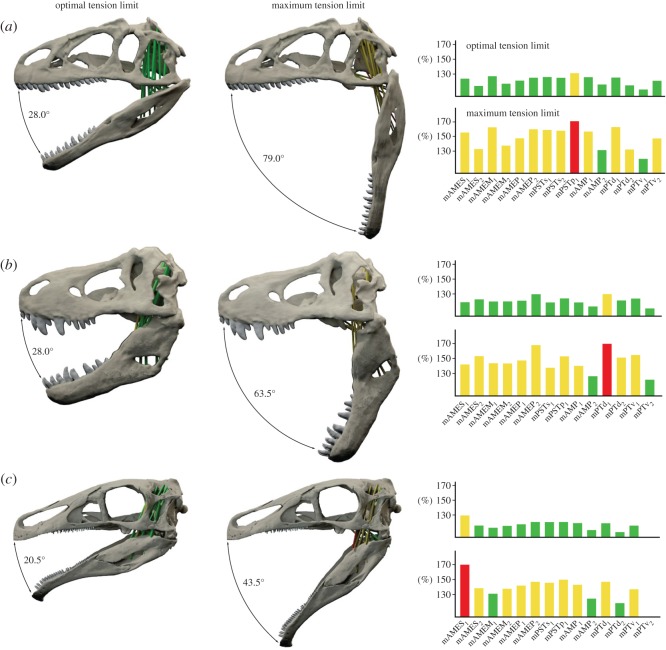
Gape angles at optimal and maximum tension limit for (*a*) *Allosaurus fragilis*, (*b*) *Tyrannosaurus rex* and (*c*) *Erlikosaurus andrewsi* with muscle resting length at a gape angle of 3.0°. Bar diagrams show strain factors of individual muscles at optimal and maximum tension limit. Muscle abbreviations as in table 1.

**Figure 8.**
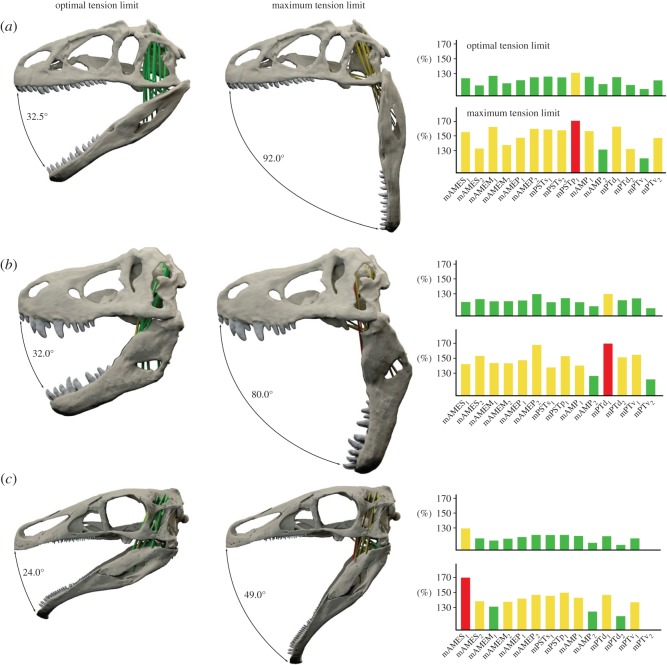
Gape angles at optimal and maximum tension limit for (*a*) *Allosaurus fragilis*, (*b*) *Tyrannosaurus rex* and (*c*) *Erlikosaurus andrewsi*with muscle resting length at a gape angle of 6.0°. Bar diagrams show strain factors of individual muscles at optimal and maximum tension limit. Muscle abbreviations as in table 1.

Accordingly, the optimal tension limit is reached at a jaw gape of 28.0° (resting length at 3.0°) and at 32.0 and 32.5° (resting length at 6.0°) in *Allo*. *fragilis* and *T*. *rex* (figures 6, 7 and 8). In comparison, the optimal gape angle is considerably lower in *E*. *andrewsi* with 20.5 (resting length at 3.0°) and 24.0° (resting length at 6.0°). Results for jaw gapes at the maximum tension limit are more distinct with 79.0° (resting length at 3.0°) and 92.0° (resting length at 6.0°) in *Allo*. *fragilis* and 70.5° (resting length at 3.0°) and 80.0° (resting length at 6.0°) in *T*. *rex* (figures 6–8). Again, *E*. *andrewsi* recorded lower maximum gape angles with 43.5° (resting length at 3.0°) and 49.0° (resting length at 6.0°; figures 6–8).

## 4. Discussion

In their seminal works on muscle fibre architecture, Huxley & Niedergerke [50] and Huxley & Hanson [51] established the relationship between the length and arrangement of muscle filaments and muscle performance. Since then, their cross-bridge model of sliding actin and myosin filaments during muscle contraction has been widely accepted and associated length changes of muscle fibres have been studied in great detail [52,53]. It is generally assumed that highest tetanic tension can be achieved in a range up to 30% length changes and a maximal tetanic tension limit occurs at 70% length changes [30,31]. However, these relationships have rarely been tested *in vivo* for complete muscle lengths. Based on theoretical calculations a maximum strain of 140% was suggested before irreversible damage occurs in mammalian jaw adductor muscles [25]. Experimentally derived measurements, however, indicate maximal possible strain factors up to or slightly above 170% are possible, but with the majority of values well below this limit in mammalian taxa [48,54].

Published measurements on maximum gape angles in extant vertebrates, and particular in birds and crocodilians, are rare. Experimental results show that mandibular angles lie between 25° and 35° but can reach angles of around 40° in birds [55–58]. When a maximum muscle strain factor of 170% is applied, the data presented here for *B*. *buteo* would result in a similar possible gape angle of 43.0° for a muscle resting length set at 3.0°. For resting lengths at larger gape angles, the results from this study are moderately higher with up to 50.5°. However, despite the fact that actual gape can be increased in birds because of the kinetic coupling of the upper and lower beaks, the postorbital ligament considerably restricts theoretically possible gape angles [59].

In comparison, data for crocodilian jaw kinematics reveal a wide range of gape angles in *Caiman* *crocodilus*, ranging from 25° to 35° when biting and crushing prey, to a maximum of 45° to 50° when transporting prey [60]. Very similar maximum gape angles (43.5° and 49.5°) were found here for *Alli. mississippiensis* for muscle resting lengths at gapes of 3.0° and 6.0°, respectively. Further experimental data for *Alli*. *mississippiensis* obtained through biomechanical modelling and experimentally derived length-tension curves [49], recorded strain factors between 110 and 151% at a gape angle of 30°. The strain analyses presented herein recorded comparable strain values between 104 and 147% (resting length at 3.0°) and between 103 and 140% (resting length at 6.0°). This suggests that the resting lengths at gape angles between 3.0° and 6.0° approach realistic values.

In living vertebrates muscles are often not simple and straight point to point connections as modelled here, but would curve around other muscles and bony structures or could attach to connective tissue. This could potentially increase or decrease the muscle length affecting muscle strain factors. Similarly, the microstructure of muscle bodies, such as fibre length, can partially influence the strain capability of muscles. However, despite these simplifications, there is a good correspondence between gape angles obtained for the extant taxa in this study and the (admittedly limited) number of published data. Furthermore, the results show a consistent strain-gape pattern regardless of resting length within the individual taxa. Therefore, the comparative approach of this method further ascertains meaningful results.

Different feeding styles and dietary adaptations have been suggested for the three theropod taxa in this study. While it has been assumed that *T*. *rex* relied on its powerful bite and robust, conical teeth in a puncture-and-pull fashion to crush bone and soft-tissues, the comparably weak muscle-driven bite in *Allo. fragilis* was used in combination with the neck musculature in a strike-and-tear mode to attack prey [40,41]. In order to be able to hunt prey in such a manner, *Allo*. *fragilis* possessed a jaw joint configuration which allowed wide gapes without the risk of dislocation [61], but would also require a muscle arrangement to permit large gape angles. Results of this study lend support to this assumption. Among the studied taxa, *Allo*. *fragilis* recorded the highest gape angle (79.0°–92.0°) when reaching the maximum tension limit, and therefore considerably larger than *T*. *rex* (63.5°–80.0°). However, both theropods reached the optimal tension limit at gape angles of 28.0° and 32.5°, which would allow high muscle efficiency within this range. In *T*. *rex*, however, the muscle strain curves for the majority of the different muscles lie on a narrow trajectory (figure 6*c*,*d*). This suggests that these muscles had a homogenous muscle performance and provided a sustained bite force, as necessary to crush bone and dismember prey.

The therizinosaurian *E*. *andrewsi* stands in stark contrast with other theropods because of its unusual cranial anatomy, which includes an edentulous premaxilla and small, densely packed, leaf-shaped teeth [44]. These anatomical modifications are thought to represent adaptations to an herbivorous diet, and biomechanical models suggest that *E*. *andrewsi* recruited the postcranial musculature to compensate for low bite forces to crop foliage and strip leaves of branches [37]. The data presented here shows that *E. andrewsi* could achieve only a comparably low gape of 43.5°–49.0° before reaching the maximum tension limit, which is consistent with the inferred feeding style. In extant mammals, herbivorous taxa generally have a distinctly smaller maximum gape than carnivores [25]. In the carnivorous grasshopper mouse (*Onychomuys* *leucogaster*) and the granivorous deer mouse (*Peromyscus maniculatus*), two sympatric and closely related murid species, dietary specialization is reflected by gape angle [62], and the same pattern of niche separation appears to apply to the theropods in this study. However, while carnivorous and herbivorous taxa show distinct differences in gape angle and muscle strain trajectories, there is no consistent pattern regarding the strain of individual muscle groups (table 3). While parts of the m. pterygoideus ventralis (m. PTv) are generally among the muscles that show the least strain, different muscle groups were found to experience highest strain across the studied taxa.

**Table 3.** Muscle strain factors at a gape angle of 60.0° with a resting length at 3.0° exemplarily for all studied taxa. (Highest values for each taxon shown in bold, lowest values shown in italics. Although actual strain values change with resting length, the same muscles show the minimum and maximum strain within one taxon, but not across all taxa. All values in per cent. Muscle abbreviations as in table 1. Subscript number indicates rostral (1) and caudal (2) part of muscle.)

muscle	*Allosaurus fragilis*	*Tyrannosaurus rex*	*Erlikosaurus andrewsi*	*Buteo buteo*	*Alligator mississippiensis*
mAMES_1_	146.41	137.80	**194**.**27**	134.02	**193**.**49**
mAMES_2_	127.85	147.22	150.08	127.53	128.00
mAMEM_1_	152.50	137.94	139.26	—	178.43
mAMEM_2_	131.47	138.64	146.25	—	**179**.**92**
mAMEP_1_	140.52	141.19	151.58	137.02	167.07
mAMEP_2_	150.33	**160**.**17**	**159**.**52**	130.47	160.57
mPSTs_1_	149.74	133.60	156.54	132.45	155.60
mPSTs_2_	148.82	—	—	132.72	—
mPSTp_1_	**159**.**85**	146.94	162.26	**163**.**31**	150.71
mPSTp_2_	—	—	—	152.11	—
mAMP_1_	148.29	134.57	152.33	147.82	137.13
mAMP_2_	126.47	*123.35*	—	155.14	125.64
mPTd_1_	**152**.**62**	**162**.**25**	*131.75*	135.27	136.25
mPTd_2_	*127.51*	145.42	162.55	**204**.**64**	*112.90*
mPTv_1_	*116.17*	148.62	*121.15*	*119.18*	*109.96*
mPTv_2_	140.09	*119.73*	146.69	*100.79*	149.94

## 5. Conclusion

The analyses of musculoskeletal structures and muscle strain trajectories presented herein not only demonstrate distinct differences in the feeding styles of theropod dinosaurs, but also confirm previous results and assumptions on dietary specialization. Although the simplified models as applied here do not take into account the internal structure of muscles, it is possible to separate dietary niche adaptations. While the method presented here is used in the context of investigating optimal and maximum jaw gape in extinct taxa, there exists the possibility to apply it in a hypothesis testing approach to evaluate the likelihood of different muscle reconstruction and myological configurations and interactions. Using the freely available software Blender further makes this a versatile approach to analyse three-dimensional models and to create numeric and graphical results.

## Data accessibility

Electronic supplementary material has been deposited in Dryad: (http://dx.doi.org/10.5061/dryad.c75j9).
